# Molecular and Mechanical Mechanisms of Calcification Pathology Induced by Bicuspid Aortic Valve Abnormalities

**DOI:** 10.3389/fcvm.2021.677977

**Published:** 2021-05-26

**Authors:** Hail B. Kazik, Harkamaljot S. Kandail, John F. LaDisa, Joy Lincoln

**Affiliations:** ^1^Department of Biomedical Engineering, Marquette University and Medical College of Wisconsin, Milwaukee, WI, United States; ^2^Cardio Consulting, Warwick, United Kingdom; ^3^Division of Cardiovascular Medicine, Department of Medicine, Medical College of Wisconsin, Milwaukee, WI, United States; ^4^Section of Pediatric Cardiology, The Herma Heart Institute, Children's Wisconsin, Milwaukee, WI, United States; ^5^Department of Pediatrics, Medical College of Wisconsin, Milwaukee, WI, United States

**Keywords:** hemodynamic, biomechanic, calcific aortic valve disease (CAVD), wall shear stress, fluid-structure interaction simulation

## Abstract

Bicuspid aortic valve (BAV) is a congenital defect affecting 1–2% of the general population that is distinguished from the normal tricuspid aortic valve (TAV) by the existence of two, rather than three, functional leaflets (or cusps). BAV presents in different morphologic phenotypes based on the configuration of cusp fusion. The most common phenotypes are Type 1 (containing one raphe), where fusion between right coronary and left coronary cusps (BAV R/L) is the most common configuration followed by fusion between right coronary and non-coronary cusps (BAV R/NC). While anatomically different, BAV R/L and BAV R/NC configurations are both associated with abnormal hemodynamic and biomechanical environments. The natural history of BAV has shown that it is not necessarily the primary structural malformation that enforces the need for treatment in young adults, but the secondary onset of premature calcification in ~50% of BAV patients, that can lead to aortic stenosis. While an underlying genetic basis is a major pathogenic contributor of the structural malformation, recent studies have implemented computational models, cardiac imaging studies, and bench-top methods to reveal BAV-associated hemodynamic and biomechanical alterations that likely contribute to secondary complications. Contributions to the field, however, lack support for a direct link between the external valvular environment and calcific aortic valve disease in the setting of BAV R/L and R/NC BAV. Here we review the literature of BAV hemodynamics and biomechanics and discuss its previously proposed contribution to calcification. We also offer means to improve upon previous studies in order to further characterize BAV and its secondary complications.

## Introduction

Bicuspid aortic valve (BAV) is the most common congenital heart malformation with an estimated prevalence of 1–3%, and a male predominance of 3:1 (1). As compared to a normal tricuspid aortic valve (TAV) containing three leaflets (or cusps), the BAV forms with only two functional cusps as the result of abnormal valvulogenesis (2). The abnormal structural geometry and resulting adverse hemodynamic environment associated with the BAV offers complexity to the disease that has yet to be fully characterized, but likely has a negative influence on the structure-function relationships normally exhibited by the TAV.

**Figure 1:**
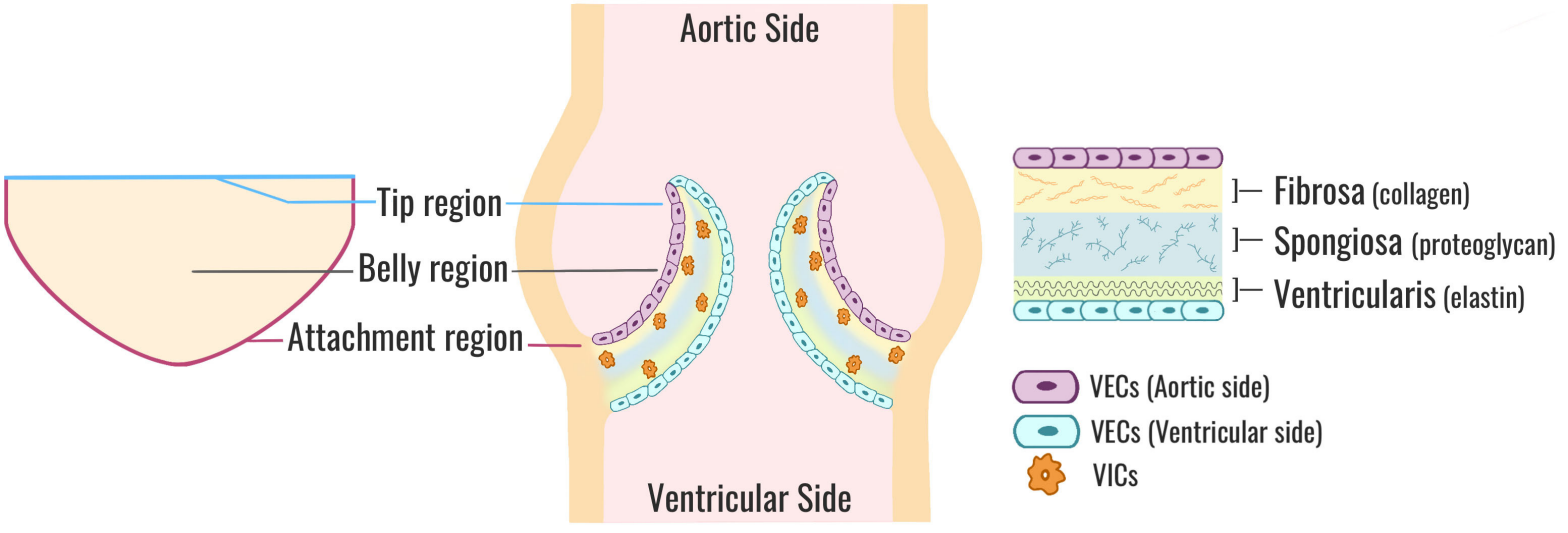
Aortic valve cusp structure. A cross-sectional view of a normal TAV (center). Each valve cusp is comprised of a highly organized ECM (right) stratified into three layers: a collagen-dense fibrosa layer (aortic side), an elastin-rich ventricularis layer (ventricular side), and a spongiosa layer sandwiched in-between comprised mostly of proteoglycans. VICs are situated within the core of each cusp and maintain ECM synthesis and homeostasis. VECs form a protective monolayer encapsulating the entire cusp. A flattened perspective of a single aortic valve cusp (left) illustrates the locations of the tip, belly, and attachment regions. TAV, tricuspid aortic valve; ECM, extracellular matrix; VICs, valve interstitial cells; VECs, valve endothelial cells.

The TAV is an avascular structure connected to the aortic root, containing three semilunar cusps that open and close to maintain unidirectional forward blood flow from the left ventricle to the aorta. The cusps are named according to their location relative to the coronary artery ostia: the left coronary (L), right coronary (R), and non-coronary (NC) (3). Each cusp is comprised of three highly organized layers of extracellular matrix (ECM), which help the valve withstand a range of hemodynamic forces as it opens and closes over 100,000 times a day (1) (Figure 1). The fibrosa layer is situated on the aortic side of the cusp and enriched with circumferentially-aligned collagen fibers that provide structural integrity to the valve. On the opposite surface is the ventricularis layer, containing radially-aligned elastin fibers to facilitate cusp motion during valve opening and recoil during valve closure. A proteoglycan-rich spongiosa layer is situated between the fibrosa and ventricularis, and provides deformability to the cusps as well as lubrication to adjacent layers (1). The ECM structure is synthesized and maintained by valve interstitial cells (VICs) that reside within the core of the cusps as quiescent fibroblast-like cells in the absence of disease. Encapsulating the valve cusp is a single layer of valve endothelial cells (VECs) between VICs and the hemodynamic environment (1). It has been shown previously that although VICs and VECs have minimal physical contact *in situ*, the two cell populations molecularly communicate through paracrine signaling to maintain ECM homeostasis and prevent disease (4–7). Previous mechanobiology studies have additionally demonstrated that VECs are mechanosensitive, allowing them to sense and respond to mechanical stimuli from the hemodynamic environment (8, 9). Together, the ECM and cellular components of the valve create an integrated and balanced connective tissue that responds to mechanical stimuli from the hemodynamic environment to maintain normal valve structure and function throughout life.

**Figure 2:**
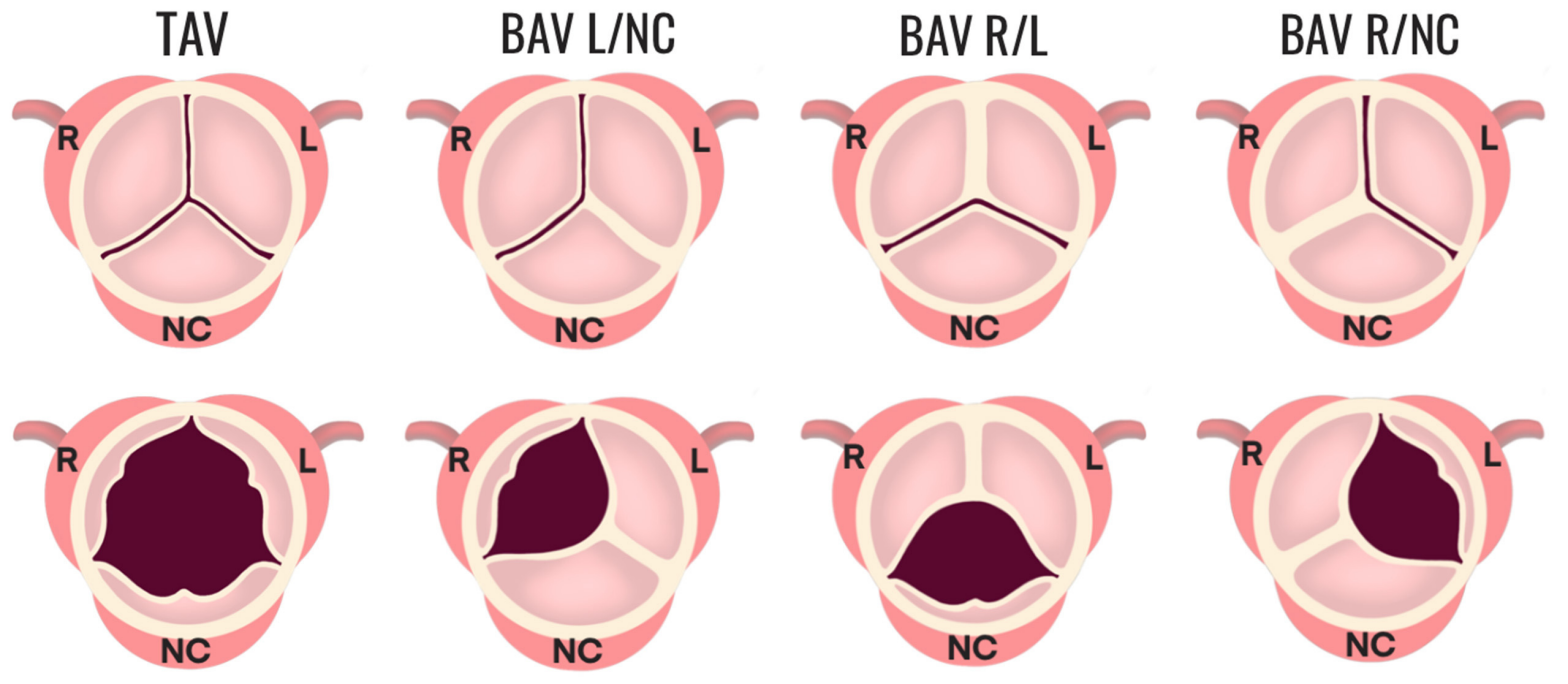
Morphologic phenotypes in BAV. The configuration of a normal TAV is shown compared to three configurations of BAV Type 1 (one fibrous raphe). BAV R/L is the fusion between the R and L coronary cusps and is the most common, accounting for 80% of Type 1. BAV R/NC is the fusion between the R and NC cusps (occurring in 17% of Type 1 cases), and BAV L/NC is the fusion the between L and NC cusps (~1%). BAV, bicuspid aortic valve; R, right; L, left; NC, non-coronary.

In cases of abnormal valve development, two of the three cusps fuse, leading to a bicuspid anatomy with unequal sized cusps that often contain a region of fibrous thickening at the fusion site, known as a raphe (10). The bicuspid aortic valve (BAV) exists in different morphologic phenotypes (Figure 2), which have been classified by different naming systems. Under the Sievers classification system, BAV morphotypes are categorized by the number of fibrous raphes present as well as the cusps that are fused together. Based on the number of raphes, BAV is classified as: Type 0 (no raphe present), Type 1 (one raphe present), and Type 2 (two raphes present). Types 1 and 2 are further categorized on the basis of cusp fusion relative to the coronary artery origins, where R/L is the fusion between right and left coronary cusps, R/NC is the fusion between right and non-coronary cusps, and L/NC between the left and non-coronary cusps (2, 11) (Figure 2). Type 1 occurs with a frequency of 90% of all BAV cases where R/L is the most common configuration (accounting for 80% of Type 1), followed by R/NC (17%), and L/NC (~2%) (11).

A genetic etiology of the BAV structural malformation is widely acknowledged and supported by studies that have established the heritability of BAV as up to 89%, indicating an almost genetically determined disease (12). Despite this, the genes and mechanisms underlying the developmental origins of forming two, rather than three cusps are largely unknown and remain poorly understood. *In vivo* models corroborated by human genetic studies have identified a few candidate genes potentially involved in the causal molecular mechanisms of BAV including *NOTCH1, GATA* family members (*GATA4, GATA5*, and *GATA6*) as well as *ROBO4, eNOS, NKX2.5*, and *SMAD* (13–15).

The severity of BAV ranges from lifelong asymptomatic disease in older adults to severe complications in childhood. However, it is not necessarily the structural malformation of BAV that necessitates clinical intervention but rather the development of BAV-associated secondary complications including aortopathy (aortic dilatation, aneurysm, dissection, and coarctation) and valvulopathy (calcific aortic valve disease (CAVD), aortic stenosis, and regurgitation) (16–18). The presence of a congenital BAV malformation is a major risk factor for developing these secondary complications, and over 50% of young adults (>35 years old) with BAV develop early onset CAVD that can progress to severe aortic stenosis within 10–12 years (16, 17, 19). As a comparison, CAVD in the TAV population affects ~25% of individuals over the age of 65 with slower progression to severe aortic stenosis (20–30 years) (18, 20–22). Calcification is thought to be an active process involving inflammation, endothelial dysfunction, ECM remodeling, and VIC phenotypic changes resulting in the formation of calcium nodules preferentially on the fibrosa layer, which stiffens the valve cusps leading to stenosis (20, 22, 23). Despite a higher prevalence of CAVD in the BAV R/L configuration, calcification is more frequent and rapid in the BAV R/NC configuration, (24) yet it is unclear why.

To date, the cause of secondary complications in BAV remains unknown. Proponents of a genetic theory hypothesize that the underlying gene mutations responsible for the structural malformation and heritability of BAV are the primary contributors to associated secondary complications. However, in addition there are a growing number of studies supporting the implications of altered hemodynamic and biomechanical influences in BAV. Abnormal mechanical stimuli imposed on the valve may disrupt the normal mechanoregulation of cellular processes responsible for valve homeostasis and subsequently lead to disease. Therefore, the role of hemodynamics and biomechanics in BAV cannot be neglected in the efforts to delineate causal mechanisms of BAV-associated complications.

BAV, and associated complications present a large clinical burden for which there are inadequate treatment options currently available. Less invasive options for BAV patients with CAVD and aortic stenosis including balloon aortic valvuloplasty often lead to early symptomatic recurrence. While more invasive approaches such as transcatheter or surgical aortic valve replacement can be associated with suboptimal long-term outcomes (25, 26). Furthermore, calcification and aortic stenosis may go undiagnosed until end-stage when treatments options are further limited (27). Therefore, BAV and its complications warrant the need for novel mechanistic-based therapies that prevent or halt the progression of calcification and aortic stenosis in BAV patients. In order to do this, the field must delineate the temporal and spatial pathobiology of this multifactorial, complex disease by integrating the impact of the hemodynamic environment on molecular and cellular changes within the bicuspid valve and surrounding tissue that drive secondary complications.

## Hemodynamic and Biomechanical Influences in BAV

There is growing evidence from fluid and solid mechanics studies demonstrating the presence of an altered hemodynamic and biomechanical environment in patients with a normally functioning BAV, which may contribute to the pathogenesis of secondary complications, such as calcification at a later time (10, 13, 28–35). The indices derived from these studies describe the hemodynamic environment and mechanical stimuli imposed on normal and diseased valves at the level of orifice area, velocity jets, transvalvular pressure gradients, as well as vortical and helical structure formation (36–41). Fluid mechanics studies have also quantified indices of wall shear stress (WSS) which is defined as the frictional force exerted from blood flow, while solid mechanics studies have assessed cusp biomechanics through measurement of stress and strain (28, 29, 32, 33, 35, 42–44). However, quantification of these indices and data directly supporting a contributing role in the onset of premature calcification remain limited.

Several methods have been employed to quantify the mechanical stimuli imposed on the BAV and uncover mechanisms influencing aortic and valvular pathology in this patient cohort. In this review, the approaches have been generally classified into three types: assessment through available clinical imaging modalities (i.e., *in vivo*), numerical simulations using image-based models (i.e., *in silico*), and bench-top approaches (i.e., *in vitro, ex vivo*). The use of these methods have provided significant advancements toward our understanding of mechanical stimuli from BAV, although as discussed below, challenges remain in accurately replicating the associated complex hemodynamic and mechanical environment.

Clinical (*in vivo*) methods including echocardiography, cardiac magnetic resonance imaging (MRI), and computed tomography (CT) often serve as the basis for computational modeling, and have been used independently in the past to describe patient-specific valve morphology and valvular performance (37–40, 45–47). These studies are most often reproduced from an adult cohort with fewer studies focusing on pediatric cases. Recently, 4D flow (temporal phase-contrast MRI with three-directional velocity encoding) has been used to obtain velocity profiles on a patient-specific basis. In principle, this could overcome some of the limitations associated with the assumptions inherent in numerical modeling, but it comes at the cost of spatiotemporal resolution (48). For example, 4D flow has been shown to underestimate WSS, which has been previously related to pathology in BAV and other vascular beds (39).

Experimental techniques such as particle image velocimetry (PIV) and laser doppler velocimetry (LDV) have been used in bench-top approaches to quantify velocity jet magnitude and direction as well as turbulence characteristics like vortical and helical formation (13, 43). Bench-top approaches may provide greater consideration of tissue material properties with the use of excised aortic valves when compared to numerical modeling. However, the use of LDV in such studies for example, is limited to velocity measurements at only a single point and while PIV can provide in-plane or even 3D velocity measurements, it is often difficult to fully replicate physiologic pressure and flow waveforms, patient-specific valve anatomy, and/or compliance of the aortic root in a bench-top approach.

Numerical methods including computational fluid dynamics (CFD) and fluid-structure interaction (FSI) simulations have been applied to BAV modeling with the goal of providing high spatial and temporal resolution (28, 29, 32, 33, 42). When accurately considering the geometric modeling process (28, 49) the result can be detailed indices that are challenging to obtain from imaging alone. Despite recent capabilities in computational modeling using CFD and FSI, many prior studies have relied on idealized assumptions regarding valve geometry, boundary conditions, and/or tissue material properties. Moreover, previous BAV studies have primarily focused on modeling the BAV R/L configuration in the adult population.

Multi-disciplinary studies coupling advanced imaging methods with state-of-the-art computational modeling are underway to create even better representations of the native and pathologic hemodynamic environments of the aortic valve. These studies attempt to address the limitations of the above approaches. A recent study by Kandail et al. (50) investigated hemodynamic alterations possible with one of the most commonly implemented transcatheter aortic valve replacement (TAVR) devices used clinically. The authors reconstructed aortic geometry from clinical imaging, virtually deployed the TAVR device, and implemented physiologically representative boundary conditions while incorporating realistic material properties for the aorta and valve. Although this model was used to study valvular flow in a different setting, it demonstrated a robust approach aimed at addressing many limitations of numerical modeling that may better replicate the physiologic environment. Due to the compounding assumptions necessary for accurate modeling and complexity of BAV hemodynamics, it is imperative that future studies of BAV patients employ similar approaches rooted in realism to link indices to mechanism of pathology.

### Hemodynamic Influences on Valvular Performance in BAV

**Figure 3:**
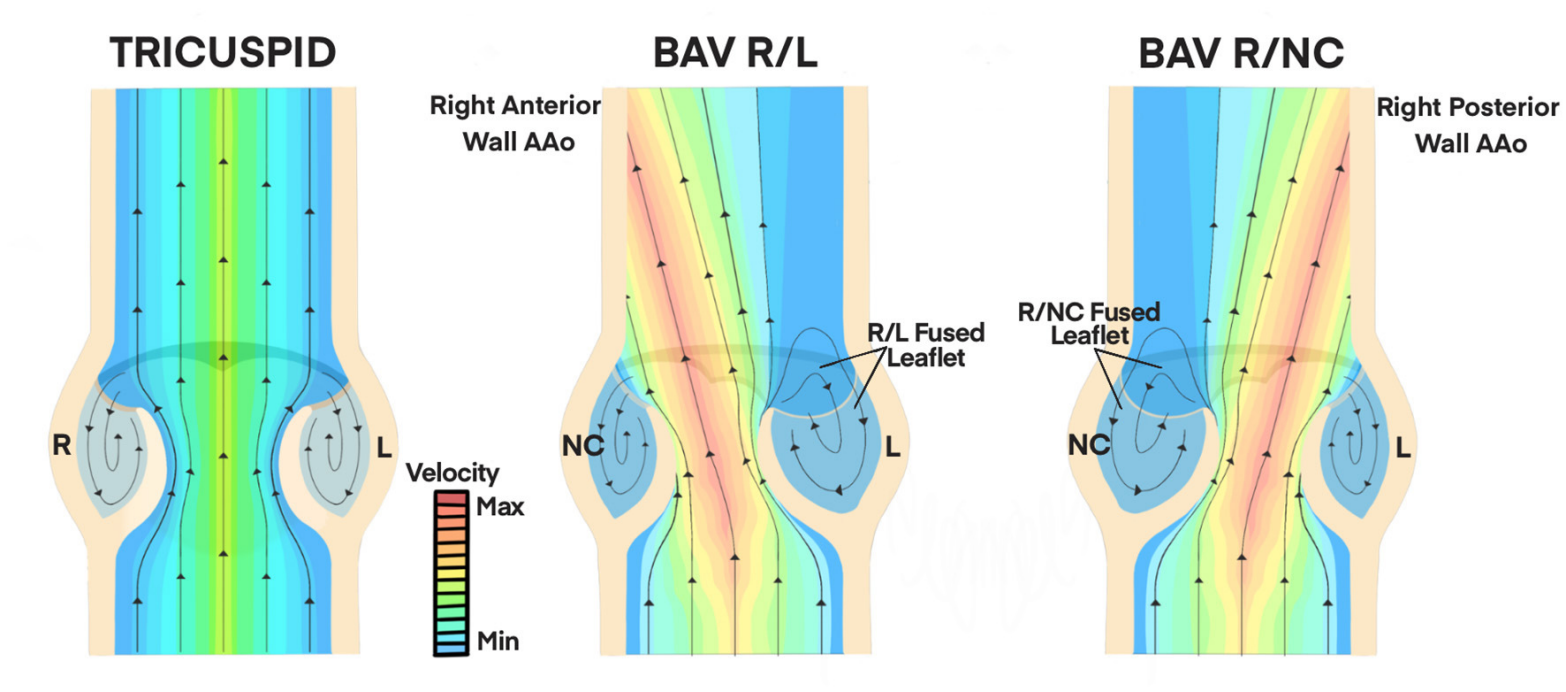
Hemodynamic Influences on Valvular Performance in BAV. A normal TAV features a centrally-aligned velocity jet through the valve orifice at physiologic magnitudes (1.1 to 2.3 m/s, minimum velocity range). Symmetrical vortices form in the cusp sinus of TAV which lead to synchronous closure of valve cusps. BAV R/L and BAV R/NC configurations have skewed velocity jets with magnitudes of 2.0 to 5.0 m/s (maximum velocity range) through the valve orifice due to asymmetric cusp geometry and impaired mobility of the fused cusp (28, 29, 32, 51–53). This is directed toward the right anterior wall of the AAo in BAV R/L and toward the right posterior AAo wall in BAV R/NC. Asymmetrical vortex formation leads to a smaller, faster vortex in the non-fused cusp sinus and a larger, slower vortex in the fused cusp sinus that extends further into the AAo (36–38, 40, 41, 47). AAo, ascending aorta; BAV, bicuspid aortic valve; R, right; L, left; NC, non-coronary.

Hemodynamic indices describing the structural geometry, valvular performance, and other hemodynamic influences have been more prominently identified in adults than pediatric patients, but nonetheless have better informed clinical decisions about BAV severity, treatment, and their implications in secondary complications (37, 38, 40, 41, 45–47). Figure 3 is a diagrammatic comparison of these features in different BAV configurations compared to a normal TAV (also see Table 1). While a normal TAV is characterized by having a wider, closer-to-round valve orifice, BAV R/L and BAV R/NC have been shown through flow visualization and imaging studies to exhibit an elliptical, clam-shell shaped valve orifice (28, 44, 45). The effective orifice area in BAV is also significantly smaller than in a normal TAV (BAV values of 1.21–2.28 cm^2^ vs. TAV of 2.90–4.26 cm^2^) (28, 29, 32, 51–53). The reduced effective orifice area ultimately results in a higher velocity jet with magnitudes reaching 2.0–5.0 m/s as compared to 1.1–2.3 m/s in a normal TAV based on computational, *in vitro*, and imaging studies (28, 29, 33, 36, 43, 54).^.^ Consequently, more severe cases of BAV are accompanied by a transvalvular pressure gradient as high as 60 mmHg in adult and pediatric BAV patients, (46) which is a frequently used clinical metric to indicate the severity of stenosis (e.g., >40 mmHg) (52). Factors including cusp geometry and stiffer tissue properties such as in cases with a raphe, are potential contributors to the impaired mobility observed in the fused cusp of BAV (2, 44). This leads to an eccentric systolic velocity jet through the valve that is skewed toward the non-fused cusp and impinges on the downstream wall of the aorta. The direction of the velocity jet, however, is dependent on the valve fusion pattern. Whereas a normal TAV geometry has a systolic velocity jet aligned centrally through the ascending aorta, the velocity jet in BAV R/L is directed toward the non-coronary cusp and impinges on the right-anterior aortic wall, while the velocity jet in BAV R/NC is directed toward the left coronary cusp and impinges on the right-posterior aortic wall (36–38, 40, 41, 47). The asymmetrical cusp geometry and jet eccentricity in BAV gives rise to abnormal vortical structures in the cusp sinuses. Computational and *in vitro* studies indicate the presence of symmetrical vortices forming in the cusp sinuses of a normal TAV subsequently lead to synchronous closure of the valve cusps (13, 29, 32, 33, 43, 53, 54). Conversely, BAV R/L appears to most often feature a small vortex forming in the non-fused cusp sinus and a larger vortex in the fused cusp sinus that extends further downstream in the aorta (13, 29, 32, 33, 43, 51, 53).

**Table 1:** Summary of hemodynamic and biomechanical influences in TAV, BAV R/L, and BAV R/NC.

**Indices**	**Normal TAV**	**BAV R/L Configuration**	**BAV R/NC Configuration**	**References**
**Hemodynamics Studies**				
Valve Orifice Shape/Size	Circular, round orifice, large valve opening area	Elliptical, clamshell-shaped orifice, reduced opening area	Elliptical, clamshell-shaped orifice, reduced opening area	(28, 36)
Systolic Jet Velocity/Direction	Centrally-aligned velocity jet at physiologic magnitudes	High velocity jet skewed towardz right-anterior wall of AAo	High velocity skewed towardz right-posterior wall AAo	(36, 41, 47, 55)
Vortex and Helical Structures	Symmetrical vortical structures in cusp sinuses Absence of abnormal helical flow downstream in AAo	Larger, low velocity vortex in fused cusp sinus; smaller, high velocity vortex in non-fused sinus Right-handed helical flow in AAo	Larger, low velocity vortex in fused cusp sinus; smaller, high velocity vortex in non-fused sinus Left-handed helical flow in AAo	(13, 29, 32, 33, 43, 53)
Cusp Wall Shear Stress (WSS)	High magnitude & unidirectional WSS on ventricularis Low magnitude & oscillatory WSS on fibrosa Magnitude gradually decreases from tip to attachment region	High magnitude & unidirectional WSS on ventricularis of fused and non-fused cusps Elevated WSS on non-fused cusp fibrosa; Sub-physiologic WSS on fused cusp fibrosa	^*^	(28, 29, 34, 42, 53, 56, 57)
**Biomechanics Studies**				
Stress/Strain	Cusp stretch and strain are greatest during diastole and in the radial direction High strain along tip region and high von Mises stress along attachment and commissural region	Increased radial strain on fused cusp while circumferential strain is similar to TAV High principal stress on the fused leaflet in attachment and commissural region	^*^	(28, 29)

*TAV, tricuspid aortic valve; BAV, bicuspid aortic valve; R, right coronary; L, left coronary; NC, noncoronary; AAo, ascending aorta*.

* *Limited data to support conclusions*.

In BAV, WSS indices are largely applicable to VECs that form an impermeable endothelium over the valve cusps. WSS indices act as mechanical stimuli that are ultimately transduced through signaling events via mechanotransduction (58). The vector components of magnitude and direction add to the complexity of some WSS indices, as both are thought to uniquely affect the process (58). The underlying mechanisms of mechanotransduction activated by WSS indices, and their implications in valve pathology are not fully understood, but studies discussed later in this review suggest that altered WSS in BAV could be the reason behind secondary complications such as premature calcification (30). Therefore, further quantifying patterns of WSS in TAV and BAV will likely help elucidate a role in the pathogenesis and acceleration of disease.

Associated literature point to several WSS indices of potential interest in BAV. WSS magnitude quantified as an average value over the cardiac cycle is most often denoted in the literature as time-averaged WSS (TAWSS), or temporal shear magnitude (TSM) (29, 58). Temporal shear gradient (TSG) is another WSS index which describes the time derivative of WSS magnitude at a given point (29, 59). WSS directionality is expressed in terms of oscillatory shear index (OSI), where an OSI of 0.0 is purely unidirectional and an OSI of 0.5 is equally bidirectional (33).

To date, a large number of studies examining indices of WSS in BAV have focused on its distribution in the ascending aorta, mainly in determining its potential role in aortic dilatation and aneurysm (36–38, 40, 41, 47, 54, 55). However, only a handful of studies have been dedicated to distinguishing WSS alterations on the valve cusps (28, 29, 32, 33, 42). Compared to the adult literature, very few studies have determined WSS abnormalities in the ascending aorta of pediatric BAV patients, (40, 45, 46, 55) and even fewer studies have attempted to characterize these alterations on the pathology of the valve cusps. Additionally, for the limited number of studies that have focused on cusp WSS in BAV, most have neglected to consider the implications of coronary blood flow on sinus hemodynamics and cusp mechanics. In fact, a recent *in vitro* study by Flemister et al. (60) using a bioprosthetic TAV highlighted the distinguishable WSS patterns in each cusp sinus when physiologic coronary flow waveforms were included (60). Their study revealed higher velocity and vorticity in the right and non-coronary sinuses compared to the left coronary sinus. Results also revealed a greater likelihood of higher WSS magnitudes in the left coronary and right coronary sinuses (60). These findings further underscore the importance of implementing coronary flow in future BAV studies to accurately replicate native flow conditions and local pressure gradients that result in distinguishable WSS patterns of different BAV configurations. It is also worth noting that studies to date have mostly characterized cusp WSS for BAV R/L (Type 1) fusion with no consideration of BAV R/NC anatomy. Though BAV R/L (Type 1) is the most common BAV configuration, the R/NC fusion pattern is associated with more frequent and accelerated progression of CAVD (24). Collectively addressing the current unmet needs for comparing BAV R/L and BAV R/NC configurations along with the inclusion of coronary flow and consideration of the pediatric population in future BAV studies may greatly enhance our understanding of BAV hemodynamics and biomechanics.

In the adult BAV population, Chandra et al. (33) employed a 2D FSI study comparing local WSS patterns on valve cusps in idealized models of normal TAV and BAV R/L. This approach imposed physiologic transvalvular pressure (diastolic/systolic ratio of 2:1) as a traction condition at the outlet. Results indicated that regardless of valve anatomy, WSS on the cusps is side-specific and site-specific. More specifically, the ventricularis is subjected to high magnitudes of WSS featuring mostly unidirectional, pulsatile flow while the fibrosa is subjected to lower magnitudes of WSS and exhibiting bi-directional, oscillatory flow. Additionally, the authors showed that WSS on any given cusp can vary along the tip, belly, and attachment regions. The BAV R/L model was marked by the existence of abnormal WSS patterns due to the cusp asymmetry. These findings were further substantiated in a follow-up study, this time using 3D FSI models in idealized adult TAV and BAV R/L and imposing a similar transvalvular pressure waveform as a traction condition at the inlet and outlet (29). In all cases, there was a similar spatial distribution of WSS on both cusp surfaces. The tip region was exposed to high magnitudes of WSS, while the belly and attachment regions were exposed to lower magnitudes. On the ventricularis, BAV R/L cusps were subjected to high WSS magnitudes on the belly and tip regions (TSM ranging between 12.3 and 44.7 dyn/cm^2^) when compared to TAV cusps (TSM from 5.8 to 19.9 dyn/cm^2^). TSG on the ventricularis was lower on the base and belly region of BAV cusps compared to TAV and greater in the tip region of BAV cusps. The BAV fibrosa was subjected to overall lower TSG than TAV with the exception of the base of the non-fused BAV cusp which exhibited higher TSG. These same regions on TAV and BAV R/L cusps experienced mostly unidirectional WSS (OSI < 0.06). While the base of the fused BAV R/L cusp experienced bidirectional WSS (OSI = 0.30), the base of the non-fused BAV R/L cusp and TAV cusps experience closer to unidirectional WSS (OSI < 0.14). Conversely, the fibrosa in all models were exposed to systemically lower WSS magnitudes than the ventricularis, but featured greater variability in regionality and directionality due to valve geometry. Compared to the fibrosa of TAV cusps (TSM from 0.8 to 3.5 dyn/cm^2^), the non-fused cusp in BAV R/L experienced elevated WSS (TSM > 3.8 dyn/cm^2^) while the fused cusp experienced sub-physiologic WSS (TSM < 1.3 dyn/cm^2^). WSS is bidirectional (OSI > 0.14) on the fibrosa of TAV cusps and BAV R/L fused cusps, but unidirectional on the fibrosa of the non-fused BAV type I (OSI < 0.03). Similar findings were attained in a highly sophisticated study conducted by Emendi et al. (28) who created a patient-specific FSI model of adult BAV R/L and compared the computationally-derived cusp WSS to values obtained by 4D flow MRI. This study reaffirmed the presence of WSS elevations on the ventricularis of BAV R/L cusps, with the non-fused cusp experiencing the highest magnitudes of WSS concentrated to the tip region (146 dyn/ cm^2^) and decreasing toward the belly and attachment regions. Further, it aligned with previous studies describing systemically lower WSS magnitude on the fibrosa of each cusp, where the fused cusp exhibited much lower WSS compared to the non-fused cusp. Although this study showed that coupling advanced cardiac imaging and computational modeling can provide realistic predictions of the *in vivo* flow environment, unfortunately results were not compared with a normal control valve to better appreciate the differences in values for WSS in diseased valves. Other computational models have outlined WSS alterations with respect to valve cusp angle and size (32) as well as applying realistic anisotropic-hyperelastic tissue properties of the valve cusps (35). These models revealed similar spatial distributions and WSS overloads in BAV models compared to TAV, although the latter study reported peak average WSS magnitudes of up to 280 and 420 dyn/cm^2^ on the cusp fibrosa and ventricularis, respectively, which are significantly higher than levels reported by other studies.

An *in-vitro* flow study with modified porcine BAV and TAV models described the shear stress on valve cusps through measurement of Reynold's shear stress, and viscous shear stress (43). These parameters ultimately indicated regions of high fluctuations, suggesting unsteady interaction between the altered flow and valve cusps. The same group demonstrated in a later study that the fibrosa of the BAV cusp experiences greater fluctuations in WSS compared to TAV cusps (13). This manifested as both magnitude variability in WSS across different cardiac cycles as well as high-frequency fluctuations within the same cardiac cycle. This was more profound on the non-fused cusp in comparison to the fused cusp due to the higher jet velocity and accompanying vortex in that sinus.

Additional studies have further compared the hemodynamic environment in normally functioning BAV and TAV models and show the presence of abnormal patterns of WSS in BAV (34, 42, 53, 56, 57). Table 1 provides a summary highlighting these key findings. Together with mechanobiology studies discussed later in this review showing the influence of WSS in mechanotransduction pathways to maintain valve health, the role of abnormal WSS and hemodynamic environment present in BAV cannot be neglected as a potential contributor to secondary complications.

### Biomechanical Influences on Valvular Performance in BAV

Besides fluid mechanics indices such as WSS, aortic valve tissue is exposed to a combination of normal, bending, tensile, and compressive stresses as the valve opens and closes during each cardiac cycle (30). Perturbations to these stresses are hypothesized to impact the function of VICs and VECs, eventually leading to valve tissue remodeling, inflammation, and calcification (30). Although several solid mechanics studies have described the stresses and strains in a normal TAV, limited data is published in the setting of BAV. The few studies (28, 29, 35, 44, 61, 62) available considering stress and strain in BAV have quantified the solid mechanics induced stress in a number of ways including: maximum in-plane principal stress, von Mises equivalent strain, and cusp stretch.

Robicsek et al. (44) was one of the first groups to investigate biomechanical stresses on cusp motion, contact, folding, and creasing in BAV. They conducted a simulation with dissected human aortic roots of BAV morphology (one 10-year-old male and two 24-year-old males) and noted excessive folding and creasing of the valve cusps. Although this was thought to be reflective of the cusp deformation stresses, the stress and strain were not quantitatively measured. Moreover, the differences between fused and non-fused cusps were not differentiated or compared to those of a normal TAV (61).

One *in vitro* study showed that while BAV and TAV cusps deformed similarly during diastole, they had significantly different deformation patterns during mid-to-late systole (61). As a result, the authors reported increased cusp strain in BAV compared to TAV during this time point, with the fused BAV cusps experiencing 24% higher strain in the radial direction (parallel to the direction of blood flow) than the normal TAV cusps. There were less significant changes in the circumferential strain at this time point.

Several computational studies modeling BAV have demonstrated the non-physiologic creasing of the conjoint cusp as well as the propensity for increased stress in all BAV cusps, with particularly high concentrations at the cusp fusion site and attachment regions (10, 29, 32, 34, 62). In contrast to the former *in vitro* study, these computational studies indicated higher stress and strain during diastole when the valves are closed rather than mid-to-late systole when the valves are open. An FSI study modeling BAV R/L and normal TAV measured the equivalent von Mises strain distribution and stretch on valve cusps throughout the cardiac cycle (29). Cusp strain increased from the base to tip regions in all valve geometries. However, concentration of strain varied spatially in the tip region between valve models: near the commissures in TAV, across the entire coaptation region of the fused BAV cusp, and in more focal areas in the tip region of the non-fused BAV cusp. Regardless of valve anatomy, cusp stretch in the radial direction was higher than in the circumferential direction and, like cusp strain, was greater during diastole and increased from the base to tip region. The BAV cusps had 3% higher radial deformation than TAV cusps with little change in the circumferential deformation. The patient-specific study by Emendi et al. (28) confirmed that principal stress was highest on both cusp surfaces of BAV R/L during diastole and revealed higher stress on the fused cusp (maximum value of 322 kPa) compared to the non-fused cusp. Another notable FSI study (35) determined that the stress imposed on fused and non-fused cusps in BAV R/L is highly dependent on cusp size and fusion angles, suggesting that cusp stresses will vary greatly from patient to patient. Table 1 summarizes the key findings from solid mechanics studies.

In addition to the hemodynamic influences in BAV, there are clearly disturbances to the biomechanical environment which may also interfere with the normal mechanosensitive regulation of valve structure and function as shown in mechanobiology studies described in the next section.

## Calcification in BAV

While physiologic stress is necessary to maintain valve homeostasis by influencing cell phenotype, gene expression, and protein activation in the aortic valve, altered or pathological stress may interfere with the normal responses to physiologic stress or even activate disease-inducing pathways, including those leading to calcification (63). Calcification is characterized by the appearance of calcific nodules on the aortic surface (fibrosa) of aortic valve cusps (8, 23, 64). While the underlying mechanisms of calcification are still largely unknown, it is thought to be an active process whereby VICs exhibit osteoblast-like characteristics in the presence of an inflammatory response, valve endothelial dysfunction, and ECM remodeling (8, 20, 23, 31). The development of calcific nodules stiffens the cusps limiting their mobility, and ultimately leading to aortic stenosis and heart failure (23, 27, 65). Currently there are no therapeutic options directly targeting calcification or preventing the formation of calcific nodules (27). The only suitable treatment for patients with severe CAVD and AS is valve replacement (27).

### Mechanisms That Prevent Calcification in Healthy Valves

VECs are a mechanosensitive cell population which utilize a variety of sensing mechanisms such as integrins and glycocalyx to transduce extracellular mechanical stimuli through various downstream signal transduction pathways and cause transcriptional changes (8). In the absence of hemodynamic disturbances, the TAV experiences physiological turnover of the valve ECM that is largely mediated by quiescent VICs. In turn, overlying VECs contribute to this homeostatic process by secreting “protective” growth factors and molecules to underlying VICs to maintain their quiescence, and prevent osteogenic-like processes (66). The anti-calcific factors emanating from VECs include endothelial NO synthase (eNOS) and transforming growth factor β1 (Tgfβ1) that have been shown to target Notch1 and Sox9 in VICs respectively, to prevent pro-calcific changes (5, 7). Additionally, the mechanosensitive capacity of VECs allows them to sense hemodynamic and biomechanical stimuli from the surrounding environment and it is thought that physiologic levels of these stimuli are required to maintain normal function and secretion of anti-calcific factors by VECs. However, in a diseased state, the stimuli imposed on VECs may be affected and potentially lead to reduced expression of anti-calcific factors, or activation of pro-calcific pathways.

### Hemodynamic and Biomechanical Influences on Calcification

VECs are known to be sensitive to WSS and strain and also play a role in preventing calcification by VICs (5, 7). However, it is unclear at this point what the downstream responses are to physiologic and pathologic stresses in VECs, and if these responses either affect the ability of VECs to prevent calcification or lead to activation of pro-calcific pathways. The mechanobiology studies reviewed below have tried to answer these questions by considering the response of VECs and VICs to both WSS and strain.

The fibrosa is known to be more vulnerable to calcification than the ventricularis (2, 60). However, in the TAV, the non-coronary cusp fibrosa is preferentially calcified whereas the fused cusp fibrosa in BAV is more prone to calcification (28–30, 60). Interestingly, both of these regions experience lower magnitude and more oscillatory WSS than regions less prone to calcification (67, 68). Additionally, while there are extensive studies in the vascular endothelium observing disease localized to regions of low and oscillatory WSS, less is known about how the valvular endothelium responds to such conditions (30, 31, 69). Despite these correlations, there are limited studies establishing the role of WSS in VECs and particularly how abnormal WSS present in BAV may contribute to valvular disease such as calcification.

*In vitro* and *ex vivo* studies have used different bioreactors to apply physiologic and non-physiologic WSS onto the valve cusps and examine the relationship between WSS and valve homeostasis (30, 31, 69–71). For example, one study applied non-physiologic WSS conditions representative of BAV (31), while other studies (69) have defined non-physiologic WSS as being outside the normal ranges for TAV. The parallel-plate system is capable of applying uniform, laminar WSS to valve tissue and earlier studies using this approach demonstrated that exposure to either steady or pulsatile WSS can affect ECM synthesis in porcine aortic valve cusps (70, 71). Sucosky et al. (69) implemented the cone-and-plate system which is capable of imposing uni-directional and more complex oscillatory WSS (69). In this *ex vivo* study, the fibrosa and ventricularis of healthy porcine aortic valve cusps were exposed to physiologic WSS (low magnitude and oscillatory WSS on the fibrosa; high magnitude and laminar WSS on the ventricularis) as well as non-physiologic WSS. To model non-physiologic WSS, the fibrosa and ventricularis were exposed to conditions normally experienced by the opposite surface. Inflammatory markers including VCAM-1, ICAM-1, TGF-β1 and BMP-2 were highly upregulated on the fibrosa when exposed to non-physiologic WSS, while expression of these markers remained relatively unchanged on the ventricularis. There were no significant changes under physiologic WSS conditions. The upregulation of pro-inflammatory pathways on the fibrosa only in response to non-physiologic WSS led this group to believe that disease initiation could be side-specific and influenced by altered WSS (69). In a follow up *ex vivo* study, improvements to the cone-and-plate system allowed both sides of the valve cusps to be exposed to different WSS conditions simultaneously (31). This design more closely replicated the *in vivo* flow environment of the aortic valve and was used to distinguish between TAV and BAV R/L configurations. FSI-derived WSS conditions of a normal TAV and BAV R/L model were imposed simultaneously on the fibrosa and ventricularis of porcine aortic valve cusps. Overall, cusps exposed to WSS conditions of a normal TAV and non-fused cusp of BAV R/L maintained valve homeostasis. However, cusps exposed to WSS conditions of BAV R/L were marked by fibrosa endothelial activation (noted by ICAM and VCAM upregulation), pro-inflammatory paracrine signaling (indicated by elevated expression of TGF-β1 and BMP-4), and ECM remodeling (via increased expression of MMP-2, MMP-9, cathepsin L, cathepsin S). Following VIC osteoblast-like differentiation, elevated levels of the bone matrix protein osteocalcin were also detected on the fibrosa and spongiosa when exposed to fused BAV WSS. Additionally, despite immunoblotting data suggesting an increase in Runx2 and α-SMA expression upon exposure to fused and non-fused BAV cusp WSS, the results were not statistically significant when compared to fresh controls (31).

eNOS is a shear-sensitive gene known to be upregulated in endothelial cells in response to laminar WSS and downregulated in response to oscillatory WSS (8). One study indicated markedly higher expression of eNOS on the ventricularis of excised calcified and non-calcified human aortic valves compared with the fibrosa and additionally that calcified valves overall expressed less eNOS than non-calcified valves (9). This agreed well with the known propensity for calcification on the fibrosa (2, 8, 30). The same group used the cone-and-plate system to impose side-specific physiologic WSS on the ventricularis and fibrosa of porcine aortic valve cusps (72). Using cGMP as a quantitative marker for NO signaling, it was noted that cGMP production increased on both surfaces when exposed to WSS compared to static conditions. cGMP was significantly higher on the ventricularis exposed to unidirectional, pulsatile WSS compared to the fibrosa which was exposed to lower and oscillatory shear stress.

Several other WSS-sensitive genes have been identified and are hypothesized to play a role in calcification, but to date remain unsubstantiated. For example, the Wnt/β-catenin pathway is a potential marker for calcification and known to be regulated by shear stress in endothelial cells, but there is no evidence to directly link this pathway and valvular WSS as causative in CAVD (8). Several miRNAs sensitive to WSS that are linked to calcification have also been identified such as: miRNA-30b which prevents signaling pathways involved in VICs osteogenic differentiation, miRNA-141 which was shown to block TGF-β1 and BMP-2 signaling, and miRNA-486-5p which is known to alter cell phenotype in response to shear stress (8, 73).

These findings suggest that altered WSS may induce inflammation, regulate endothelial function, mediate ECM remodeling, and upregulate osteogenesis-related proteins, all of which contribute to calcification in the aortic valve. See Table 2 for a summary of these findings. While many shear-sensitive markers have been identified in vascular endothelial cells and to a lesser degree in VECs, only a few studies consider these markers in response to WSS conditions of BAV. Furthermore, while BAV R/L has been represented in such studies, the BAV R/NC configuration has yet to be explored. This could be explained by the lack of data regarding WSS conditions specific to the BAV R/NC configuration.

**Table 2:** Summary of WSS and Stretch/Strain dependent marker expression in TAV and BAV.

**Markers**	**WSS-dependent Expression**	**Stretch/Strain-dependent Expression**	**References**
Inflammatory Paracrine Signaling	Altered WSS on fibrosa, but not the ventricularis upregulates TGF-β1 & BMP-2 Simultaneous exposure of ventricularis and fibrosa to BAV fused cusp WSS upregulates TGF-β1 & BMP-4	↑ BMP-2, BMP-4 expression with higher cusp stretch; preferentially expressed on fibrosa vs. ventricularis	(31, 69, 74)
Endothelial Activation	ICAM & VCAM are upregulated on fibrosa, but not ventricularis when exposed to altered WSS Simultaneous exposure of ventricularis and fibrosa to BAV fused cusp WSS upregulates ICAM & VCAM	↑ expression of VCAM-1, ICAM-1, & E-selectin in VECs when exposed to sub-physiologic and supraphysiologic strain↑ expression of VCAM-1 in VICs when exposed to sub-physiologic strain	(31, 72, 75, 76)
ECM Remodeling	Exposure to BAV fused cusp WSS upregulates MMP-2, MMP-9, Cathepsin L, and Cathepsin S	↑ expression of MMP-1, MMP-2, MMP-9, cathepsin K, cathepsin S and ↓ expression of cathepsin L in response to higher cusp stretch	(31, 77)
Osteoblast-like Differentiation	Elevated osteocalcin on fibrosa upon exposure to BAV fused cusp WSS	↑ Runx2 expression at higher cusp stretch, preferentially on fibrosa	(31, 74)
NO Signaling	↑ eNOS expression on ventricularis (high magnitude & unidirectional WSS) vs. fibrosa (low & oscillatory)	^*^	(72)

* *No known reported data to support conclusions*.

*WSS, wall shear stress; BAV, bicuspid aortic valve; ECM, extracellular matrix; VECs, valve endothelial cells; VICs, valve interstitial cells; NO, nitric oxide; eNOS, endothelial nitric oxide synthase; BMP, bone morphogenic protein; TGF-β, transforming growth factor beta; ICAM, intercellular cell adhesion molecule; VCAM, vascular cell adhesion molecule*.

Findings from biomechanics studies show that the array of stress and strain experienced by a normal TAV is significantly altered in BAV (28, 29, 35, 61). The following mechanobiology studies suggest that deviations from physiologic strain may contribute to calcification through the increase in pro-inflammatory markers and by mediating ECM remodeling in the aortic valve (74–77). These findings are based on *ex vivo* and *in vitro* methods utilizing uniaxial and biaxial stretch simulations to replicate native aortic valve deformations. While both physiologic and non-physiologic strain has been examined, these conditions are not BAV-specific and therefore do not directly address the role of strain in BAV complications.

An *in vitro* study described the role of mechanical strain in porcine VECs when exposed to cyclic equibiaxial strains of 0–5, 0–10, and 0–20% for 24 h, where 10% was considered to be in the physiologic range (76). Cyclic strain was shown to regulate pro-inflammatory markers including VCAM, ICAM, as well as endothelial leukocyte adhesion molecule (E-selectin) in response to strain. Significant upregulation of VCAM-1, ICAM-1, and E-selectin were measured at cyclic strains of 0–5% and 0–20% compared to 0–10% strain and controls. The VEC monolayer in 0–5% and 0–20% strains also presented with decreased integrity and increased cell death compared to 0–10% strain. A follow-up *in vitro* study investigated how mechanical strain affects inflammatory response in porcine VICs at cyclic strains of 0%, 5%, 10%, 15%, and 20% (75). At static (non-physiologic) culture conditions, VICs highly expressed VCAM-1 as well as multiple other inflammatory markers after 2 hours. Upon exposure to strain, levels of these markers dropped, however, VCAM-1 remained upregulated in cells strained at 5% and 10% compared to 15% and 20%. An *ex vivo* study demonstrated that cyclic stretch can regulate expression of BMPs in porcine aortic valve cusps which were exposed to 10% and 15% stretch for 3, 7, and 14 days in osteogenic media containing TGF-β1 (74). After 3 days, BMP-2, BMP-4, and Runx2 were preferentially expressed on the fibrosa in response to 10% and 15% stretch compared to the ventricularis and also greater at 15% stretch compared to 10%. Additionally it was observed that aortic valve cell apoptosis increased when stretched at 15% after 7 days compared to 10% stretch. Introducing BMP antagonist noggin to the media blocked osteogenesis-related activity in the cusps including Runx2, alkaline phosphate, and osteocalcin expression. Elevated cyclic stretch was shown in another study to alter ECM remodeling in aortic valve cusps (77). In this study, circumferentially-aligned porcine aortic valve cusps were stretched to 10% (considered physiologic in this study) and 15%, 20% (pathological) in a tensile stretch bioreactor for 24 and 48 h. Expression of ECM remodeling enzymes MMP-1, MMP-2, MMP-9 and cathepsin L, S and K were quantified in addition to cell proliferation and apoptosis. Cusps exposed to cyclic stretch of 10% yielded expression levels of ECM remodeling enzymes similar to controls. At 15% stretch, cusps demonstrated upregulation MMP-1, MMP-2, MMP-9, cathepsin K, S expression whereas cathepsin L expression was downregulated. There was similar trend seen at 20% stretch but was less prominent. An increase in cellular proliferation and apoptosis at 15% and 20% cyclic stretch suggested that strain of this magnitude disrupted normal valve homeostasis. The findings from these studies are summarized in Table 2.

Mechanobiology studies observing strain in the aortic valve have illustrated its ability to regulate processes including inflammation and ECM remodeling (74–77). The physiologic and non-physiologic strain conditions employed in these studies were not BAV specific and therefore may not fully reflect the extent that strain plays a role in BAV. Additional studies imposing the strain conditions experienced by BAV may provide greater insight to the mechanisms of BAV associated complications.

It is important to note that although mechanobiology studies have provided insight to many differentially expressed genes and pathways responsive to WSS and strain, evidence to implicate them as causal mechanisms in BAV-associated complications is lacking. Further, while these mechanisms have been identified in *ex vivo* and *in vitro* studies, it is imperative that future studies attempt to recapitulate the hemodynamic and biomechanical environment using *in vivo* BAV models in order to corroborate the findings from *in vitro* and *ex vivo* studies.

## Discussion

BAV and its secondary complications pose a significant health care burden for which there are limited therapeutic options. Findings from hemodynamics studies have shown that BAV experiences a range of abnormalities compared to TAV including skewed velocity jets with higher magnitude, a greater transvalvular pressure gradient, asymmetrical vortical structures in the cusp sinuses, and abnormal helical formation in the ascending aorta. BAV is also subjected to abnormal WSS patterns which are side-specific and site-specific. Importantly, the fibrosa of the fused cusp (most prone to calcification) experiences lower magnitude and more oscillatory WSS than the fibrosa of TAV cusps. Additionally, the ventricularis in BAV is subjected to overall higher magnitude WSS compared to TAV cusps. Biomechanics studies comparing the structural-induced alterations in BAV have shown increased strain and radial cusp stretch compared to TAV with little difference in circumferential stretch. Mechanobiology studies have revealed that the WSS and strain imposed on VECs can regulate inflammatory response, endothelial function, ECM remodeling, and VIC phenotype which are all contributing processes leading to calcification.

Although we have a greater understanding of the hemodynamic and biomechanical environment of BAV from the clinical imaging studies, numerical modeling, and bench-top approaches employed in the studies above, the data available are not currently sufficient to directly show causality for BAV-associated secondary complications such as calcification. Deepening our understanding of these complex mechanisms will require more comprehensive studies using advanced cardiac imaging modalities and more realistic FSI simulations. For example, if the lack of data surrounding the BAV configuration most prone to developing calcification (BAV R/NC) were more extensively studied, resulting data may provide insight related to the underlying mechanisms of CAVD. Similarly, the pediatric population should now be studied using state-of-the-art computational studies to describe the hemodynamic and biomechanical environment of BAV in its earlier stages before the onset of calcification. Inclusion of these aspects in future investigations may elucidate the mechanisms responsible for secondary complications in BAV.

## Author Contributions

HKaz performed literature review and drafted the article. HKan, JLi, and JLa contributed to revising and editing the manuscript. All authors read and approved the final manuscript.

## Conflict of Interest

The authors declare that the research was conducted in the absence of any commercial or financial relationships that could be construed as a potential conflict of interest.
